# Bacteriophage: Time to Re-Evaluate the Potential of Phage Therapy as a Promising Agent to Control Multidrug-Resistant Bacteria

**Published:** 2012

**Authors:** Masoud Sabouri Ghannad, Avid Mohammadi

**Affiliations:** 1 *Department of Microbiology, School of Medicine, Hamadan University of Medical Sciences, Hamadan, Iran*

**Keywords:** Bacteria, Bacteriophages, Drug resistance, Therapeutics

## Abstract

Nowadays the most difficult problem in treatment of bacterial infections is the appearance of resistant bacteria to the antimicrobial agents so that the attention is being drawn to other potential targets. In view of the positive findings of phage therapy, many advantages have been mentioned which utilizes phage therapy over chemotherapy and it seems to be a promising agent to replace the antibiotics. This review focuses on an understanding of phages for the treatment of bacterial infectious diseases as a new alternative treatment of infections caused by multiple antibiotic resistant bacteria*. *Therefore, utilizing bacteriophage may be accounted as an alternative therapy. It is appropriate time to re-evaluate the potential of phage therapy as an effective bactericidal and a promising agent to control multidrug-resistant bacteria.

## Introduction

Bacteriophages (or phages) were discovered in 1915 and 1917 by Frederick Twort and Félix d’Herelle respectively. However, d’Herelle was the first scientist who applied the term “*bacteriophage*”; a microbe that has the potential to attack bacteria and kill them. He was also the first to report that the agent of bacterial death was in fact a virus (1).

Phages are reported as the most abundant organisms on earth (2) and are ubiquitous in the nature. More than 5000 classified bacteriophages are known (3). Phages are easily identified in the water sources, sewage soil and even ocean depths (4) and that can be isolated easily. In the first step of isolation, the sample assumed to contain specific phages against the target bacteria is placed in a suitable salt solution and the supernatant is then filtered with appropriate filter to remove bacteria. The second step is to examine the culture supernatant of suspected bacteria. If the bacteriophage which is specific to the bacteria appears, plaque formation will occur in the latter days (5).

For almost every bacterial species, there exists at least one phage that can exclusively infect and eventually destroy that particular bacterial group. Based upon the replication methods, phages are classified as either lytic or lysogenic. A lytic phage replicates in bacterial host and destroys its host in a process but a lysogenic phage inserts itself into the genome of its bacterial host and establishes a stable position in the bacterium which has been infected (6). Lysogenic phages transfer genes which express toxin proteins or pathogen factors among bacterial species (7).

After discovery, bacteriophages were the subject of multiple researches for treating the bacterial diseases, such as dysentery (8). In the 1920s and 1930s Lilly and Squib worked on preparation a bacteriophage for the treatment of *Staphylococcus *infections. Several studies which has been performed showed that artificial skin included bacteriophage inhibited the infection of skin transplantation (7- 9). However, a number of factors including antibiotic discovery caused the decreased use of bacteriophage for medical applications. The antibacterial effect of phages accounts as a function of their replication in the host bacteria and as long as their host persists, they will continue to grow exponentially. Only a low concentration of bacteriophage can grow in the bacterial culture to a degree that they can inhibit the growth of the host bacteria (1, 10). However, after killing the host bacteria, the titre of infectious bacteriophages will decrease (11). Recontamination by bacteria increases bacteriophage population so that bacteriophages are able to prevent the infection frequently (12) and the prevalence of resistance in bacteriophages are less than bacteria (13). Specific receptor of a phage is necessary for binding of phage to the bacterial surface. These groups of viruses are not able to enter eukaryotic cells including that of human and animal because of lacking the phage’s receptor (11). 

The anti-bactericidal effects of bacteriophage are generally studied in 2 divisions, active and inactive. In active replication the number of bacteriophages is lower than bacteria but they attach to bacteria and replicates efficiently. In the inactive replication, the number of primary bacteriphages is higher than bacteria so that all bacteria are infected and lysed by primary bacteriophages (14-20). 

**Multidrug-resistant pathogenic bacteria and phage therapy**

Despite great advancements in antimicrobial therapy, the most serious challenge is the appearance and spread of multidrug-resistant pathogenic bacteria. Moreover, treatment of bacterial infections is still cost consuming. Some sophisticated therapy which weaken the patients’ immune system and make them more susceptible to nosocomial infections, highlights the importance of various prevention methods. Since bacteria have evolved the mechanisms which enable them to grow in the presence of antibacterial agents, elimination of the nosocomial infections is becoming increasingly more difficult resulting in an alarming rate of changing and developing infections.. Bacterial resistance to antibiotics and other aspects of antibiotic consuming like as the risk of breast cancer in women (21) necessitates new disinfecting policies. Several reports have been published which address perspectives of using phages against bacterial infections. They explain application of living phages can cure infectious diseases which are caused by Gram-negative bacteria, such as *Escherichia coli*, *Pseudomonas aeruginosa*, *Acinetobacter baumannii*, *Klebsiella pneumoniae*, *Vibrio vulnificus*, *Salmonella* spp and also Gram-positive bacteria, such as *Enterococcus faecium* and *Staphylococcus aureus* (22-27). 

As antibacterial agents, bacteriophages are easy to distinguish from different aspects including virolysin producing, antimicrobial peptide encoding, delivering system for genes encode antimicrobial agents and infecting sensitive bacteria as a living phage. In this study, we attempt to have a comprehensive review of all aspects of usage of phages which seem important.

***Treatment by using virolysin***

Virolysins are significant kind of bacterial cell wall hydrolases which degrade the peptidoglycan of the bacterial cell wall and help to nascent phages release. Lytic double stranded phages are responsible for encoding virolysins as the final stages of the phage lytic cycle (28). The hydrolysis process of the bacterial cell wall by virolysin consists of 2 steps: the first step is to bind to the sites on bacterial cell wall and the second is to cleave the peptidoglycan bonds (29). Hence, virolysins are the killer of Gram positive bacteria and narrow spectrum of lytic enzymes. virolysins have been used for therapy of a variety of pathogen bacteria such as: *Staphylococci*, group A *Streptococci*, * Streptococcus* *pneumoniae*, *Enterococcus  faecium*, *Bacillus* *anthracis*, *S.* *pneumoniae* and *Clostridium* bacteria (30).

There are some positive points in using virolysins as antibacterial therapy agents:

1. They are an acceptable alternative for treatment of antibiotic resistance bacteria.
2. Immunogenicity is not a concern to their effectiveness (28).
3. Different researches have demonstrated that enzyme resistant strains of pathogenic bacteria are not developed by using sub-lethal doses of virolysins (31).
4. Effective dose of virolysin is noticeably low (milligrams or even micrograms per litre). Consequently, allergic responses and neutralizing by immune system are prevented (32, 33).
5. On the other hand, resistance to the virolysins is unlikely because the mutations changing the bacterium cell wall would in fact kill the bacterium (34).
6. They eliminate the pathogenicity of bacterium rapidly.

As noted above virolysins seem to be a convenient option among antibacterial therapy choices and have been tested for the control of various bacterial infections such as *Entero. faecalis*, *Entero. faecium*, *Staphylococci, B. anthracis*, *Sterptococci* (group A), *Sterp. pneumoniae* and *Clostridium* (30). However, they have no effect on gram negative bacteria. In addition, their production is more expensive in comparison with antibiotics.

Another application of virolysins is using them as bacterial ghost vaccines which punch holes in the bacterial cell wall and release the cell components. Immunization by these vaccines has been shown to be protective (35, 36). 

Virolysins have other applications such as DNA extraction from bacterial cell, pathogen detection (lyses of bacterium cell wall by virolysins results in releasing numerous antigens that are detectable by appropriate antibodies) (37) and releasing recombinant proteins from bacterial cells (38).

***Antimicrobial peptides encoded by phages***

 Bacteriophages encode two types of antimicrobial peptides: lytic factors and phage tailed complexes. Lytic factors function as a kind of virolysin-holin system that induces bacteriolysis at a particular time. There are different types of lytic factors including: E lytic factor encoded by φX174, L lytic factor encoded by MS2/GA classes of RNA phages, A2 lytic factor encoded by RNA Qβ/SP phages. Phage tailed complexes identify specific receptors on the surface of bacterium, penetrate through the outer membrane and lyses the peptidoglycan, then inject the phage genome into the bacterium. The best example of phage tailed complexes is the tail of bacteriophage T4 (40-43). Since the mode of action of lytic factors is not completely understood and there are many obscure points about using them and also phage tailed complexes as alternatives to antibiotics, still further investigations seem to be necessary (44-46). 

***Phages as therapy delivery system***

Nowadays efficient viral delivery systems are developed to introduce the appropriate genome to the target cells (47). Phages can also be utilized as therapy delivery systems. In this process phages deliver genes encoding antimicrobials, or toxic antimicrobials into target bacteria (48, 49). Furthermore, filamentous phages have the ability to carry therapeutic genes into target mammalian cells. In this approach, mammalian cells are transduced by a receptor-mediated endocytosis. This process, by itself, is not an antimicrobial process but can be progressed to deliver antimicrobial genes into intracellular bacterial pathogens (50).

***Therapy using living phages***

It is necessary to note since 1919 in the former Soviet Union and Eastern Europe, bacteriophages have constantly been a novel target for antibacterial intervention and have been clinically used for the treatment of resistant bacterial infections.

Nevertheless, since the initiation of antibiotics consumption in the western countries, the scientists have not considered the use of phages for the treatment of bacterial infectious diseases (51). Not all phages are appropriate for phage therapy. Lytic phages that infect their hosts rapidly replicate and produce a lot of progeny and ultimately lyse the bacteria which are preferred to be compared with the phages that integrate their genome into the bacterial genome (52).

*Experiments of phage therapy in humans*

Before 1940, a variety of infections including: gastroenteritis, sepsis, suppurative wounds, dermatitis, osteomelitis, emphysemas and pneumonia were cured by phage therapy in humans (children and adults). In these experiments, the success rates were about 80 to 95 percent and there was no undesirable reaction (53). 

The emergence of antibiotic-resistant bacteria leads to considering phage therapy as a valuable alternative which is nowadays under investigations. For instance, in studies performed by the Institute of Immunology and Experimental Therapy, Slopek *et al* conducted a series of investigations. In the first step they evaluated phage therapy on 150 patients with suppurative bacterial infection. In 137 patients out of 150, the positive results were obtained and the successive rate was 91.3%. One year later they designed the same study on 114 children with suppurative bacterial infection. One hundred and nine (91.3 %) cases were cured by phage therapy. In another survey they investigated the effect of phage therapy on 370 cases with suppurative bacterial infection which in 342 cases (92.4%) positive therapeutic results were obtained. This survey confirmed the great effectiveness of bacteriophages in the treatment of septic infection, spontaneous or postoperative caused by pyogenic *Staphylococci*, *Escherichia*, *Klebsiella*, *Proteous* and finally *Pseudomonas *bacteria. These results were confirmed by the further investigations conducted by slopek *et al* which showed the same outcome. It is noticeable that in all of these studies the majority of the patients who were under phage therapy showed antibiotic resistant. Of the Polish Academy of Science, 550 patients with different bacterial infections involving *Klebsiella*, *Salmonella*, *Pseudomonas*, *Staphylococcus*, *Streptococcus* and antibiotic-resistant *E. coli* infections were successfully treated by phage therapy with the success rate of 75-100% (54-60). Other examples are the treatment of *Bacillus* *anthracis* and *Propionibacterium* *acnes* (responsible for emerging acne on the skin) by corresponding phages (60, 61).

*Phage therapy and limitations*

Utilizing bacteriophages as therapeutic agents has encountered human with some problems including:

1. Development of antibodies against phages.
2. Uptake and inactivation of the bacteriophage by the spleen.
3. Contamination of phage preparation with bacterial component such as endotoxins.
4. Although significant knowledge about the genetics of phages and their effectiveness is present but little is known about their behaviour in vivo particularly in humans (27).
5. Phage therapy may fail as a result of differences between physiology of bacteria that are inhabitant of human body and bacteria grown under laboratory condition (52).
6. There is no guarantee that lytic phages under laboratory condition remain lytic in human body. It may change to lysogenic phages.

1. In a conversion of lytic phage to lysogenic phase, the bacterium containing prophage will be immunized against the corresponding lytic phage and also the bacterial virulence may be altered (62).
2. Bacteriophages may encode toxins.
3. Lyses of bacteria by macrophages may result in release of vast amount of endotoxins that would be harmful for the body (63).
4. Resistance to bacteriophages may occur by different mechanisms including:

a) Omitting the specific phage receptors from the surface of the bacterium cell, 

b) Integrating genome of phage into the genome of bacterium, 

c) Loss of a specific gene which is necessary for the phage replication or assembly (64).

1. A study performed by Khajeh karamoddini *et al* showed that bacteriophage concentration, incubation duration and the method of culture on the antibacterial effects of phages are counted as other limitations of phage usage (65).

An easy way to solve many of the aforementioned problems is using products of phages instead of the whole phages. There are two examples of these products. The first one is capsid protein A2 that is derived from the RNA phage Qβ. The mode of action of A2 is similar as penicillin, and affects the synthesis of bacterium cell wall by interfering with related enzyme (66). The other example is a lysis protein called E protein of phage φX174 (a kind of DNA phages) (67).

*Advantages of phage therapy*

1. High specificity for target bacterium.
2. As long as the bacterium is present, the phage will be able to reproduce itself. Therefore several administrations are unnecessary. On the other hand, phages are self-replicating and self -limiting.
3. The receptors of phages on the bacterium cell wall are mainly virulence factors, so if a phage-resistant bacterium emerges, it would be less virulent (27).
4. Bacteriophage therapy would be useful in the allergic patients to antibiotics.
5. Bacteriophages do not infect human or animal cells. This makes them safe to be utilized clinically.
6. Because of their high specificity, bacteriophages do not affect microbial flora of the human body.
7. The frequency of phage mutation is higher than that of bacteria, so if a phage-resistant bacterium emerges, the phage responds quickly (68).

8- Compared with antibiotics, phage therapy is inexpensive (63).

9- Few side effects of phage therapy have been reported.

***Therapy using phages and antibiotics together***

The combined use of bacteriophage and antibiotics seems to be more effective than either of them alone. This kind of combined product is available in Georgia in the USA. There are some positive points in this combination such as increasing the efficiency of therapy and reducing the emergence of resistant bacteria (69).

***The prospects of bacteriophages***

Regarding the limited development of bacterial resistance to phages, bacteriophages might be recommended as valuable and maybe the only effective antimicrobial agent against some bacteria in specific situations. It is indeed time to re-evaluate the potential of phage therapy as a promising agent to control multidrug-resistant bacteria. This was an assessment of bacteriophages to control pathogenic bacteria without harmful effect for ecosystem and human life so that bacteriophages seem very promising for this purpose. However, due to the nature of phages, care should be taken in the cases of *in vivo* utilization, once this new technology is successfully and safely experimented. In spite of remarkable effects of phage application such as antibacterial, slow activity and high specificity of action, it necessitates acquiring precise knowledge of infecting agent. Also *in vivo* bactericidal effects of phages are poorly understood, so further studies have to be conducted to clarify it (70). Whether bacteriophage lytic enzymes with high efficacies and strong effects on their targets can be utilized as a drug in treatment of the broad spectrum of bacterial diseases awaits further investigation. 

## Conclusion

Nowadays an increasing inclination has been made throughout the world in field of phage technology which has led to novel findings therapy (51). The main aim of this study was to evaluate the efficacy of bacteriophage application as a new alternative bactericidal in reduction and/or elimination of multidrug-resistant pathogenic bacteria. Phage specific receptor is necessary for binding of phage to the bacteria surface and so that viruses are not able to contaminate the eukaryotic cells including human and animal because of lacking the phage’s receptor on eukaryotic cells (11). Thus bacteriophage seems harmless for human, animal and plant cells. For the above reasons a number of clinicians and scientists are interested to use bacteriophages in order to treat bacterial infections (51). Thus, phages can be considered as potential antibacterial agents. It seems that phage therapy is highlighted as the headlines of research subjects now (64).

In this study, we concluded that phage is an effective bactericidal agent and promising agent to control multi drug-resistant bacteria. It will inevitably be required to study the *in vivo* antibacterial effect of bacteriophages which may be similar or different to what might be observed *in vitro.* Thus, the detailed roles of phages to cure diseases still remains an open question which needs to be investigated and to clear the exact mechanisms by which bacteriophages can be utilized to pave the way for treatment of bacterial infection *in vivo *and to overcome some of the restrictions we are encountered.
